# Detection of Atrial Fibrillation Using Multi-Site Ballistocardiogram with Piezoelectric Rubber Sheet Sensors

**DOI:** 10.3390/s25226833

**Published:** 2025-11-08

**Authors:** Satomi Hamada, Miki Amemiya, Tetsuo Sasano

**Affiliations:** 1 Department of Cardiovascular Medicine, Institute of Science Tokyo, Tokyo 113-8519, Japan; hamamlab@tmd.ac.jp (S.H.); amemiya.miki@tmd.ac.jp (M.A.); 2 Department of Clinical Laboratory, Institute of Science Tokyo Hospital, Tokyo 113-8519, Japan

**Keywords:** ballistocardiography, atrial fibrillation, machine learning, piezoelectric sensor, arrhythmia, multi-site recording, fast Fourier transform

## Abstract

**Highlights:**

**What are the main findings?**

- An algorithm to detect atrial fibrillation was developed, which worked with a ballistocardiogram recorded using sensors placed at various locations from the head to the lumbar region.
- Combined assessment using multiple sensors improved the accuracy.

**What is the implication of the main finding?**

- This algorithm is tolerant of the misalignment of the body relative to the sensor from the head to the lumbar region.
- This model is valuable for practical applications as it accommodates body movements during sleep.

**Abstract:**

Ballistocardiography (BCG) is a noninvasive modality for detecting cardiac activity. This study developed a robust atrial fibrillation (AF) detection algorithm using multiple BCG sensors at different locations and evaluated the improvement in accuracy by combining data from multiple sensors. We recorded the BCG using a piezoelectric rubber sheet sensor and an electrocardiogram in 84 participants (29 with AF and 55 without AF) in the supine position. Four BCGs (BCG1–4) were obtained using sensors placed from the head to the lumbar region (0, 25, 45, and 65 cm from the head). The BCG signals were divided into 32 s blocks and analyzed. After applying fast Fourier transform, we input the power spectrum, focusing on frequencies below 10 Hz, into machine learning (ML) classifiers to distinguish between AF and non-AF with parameter tuning. The AdaBoost classifier for BCG2 exhibited the highest accuracy (0.88) among the ML models for each sensor. When we applied the classifier to other BCGs, it achieved accuracies of 0.92, 0.73, and 0.78 for BCG1, 3, and 4, respectively. The combined model using multiple sensors exhibited an accuracy of 0.92. The optimized model for BCG2 was robust against shifts in the sensor toward the head and lumbar directions. A combined assessment using multiple sensors improved performance.

## 1. Introduction

Atrial fibrillation (AF) is a common arrhythmia associated with an increased risk of death and many diseases such as stroke, heart failure, cognitive impairment or dementia, and so on [1]. Although AF is sometimes paroxysmal or asymptomatic, cardiovascular events occur almost as often in asymptomatic as in symptomatic patients [2]. AF is diagnosed using electrocardiography (ECG), which requires electrodes to adhere to the skin. A long monitoring period has been reported to increase the rate of AF detection [3]; however, it has also been reported that wearing ECG electrodes for a long time could cause adverse skin reactions including skin irritation and redness [4,5,6]. Patient-friendly methods have been sought to use for long-term monitoring of AF.

Ballistocardiography (BCG) is a noninvasive modality used to record subtle heartbeat-induced motions of the human body. These motions occur because of the rapid acceleration of blood movement from the heart to the great vessels [7]. Recently, several sensors for BCG have been developed [8,9]. They are typically attached to mattresses, beds, and chairs. Owing to their noncontact nature and minimal burden on the participants, their application in long-term cardiac monitoring is anticipated. Several studies have been conducted to estimate the heartbeat [10,11] and detect AF [12,13,14,15]. Koivisto et al. attempted to classify 30 and 3 min signals from 20 AF and 15 non-AF participants using a microelectromechanical accelerometer-based sensor, sensor built-in algorithms, and a random forest classifier [12]. Jiang et al. collected 24 s BCG segments from 59 patients with paroxysmal AF, yielding 1000 AF and non-AF segments, and attempted classification using an attention-based multi-scale features fusion method [13]. They also detected AF using recurrence quantification analysis on consecutive time windows of BCG segments [14]. Sandelin et al. collected data in a hospital at night from 116 patients and attempted to classify 10 min, 30 min, 1 h, and full-length recordings by inputting the features calculated using an autocorrelation algorithm into rule-based algorithms and machine learning (ML) classifiers [15]. With these studies, they aimed to detect AF in people lying in bed, used various types of BCG sensors and algorithms, and achieved an accuracy of 0.88–0.96, recall of 0.93–1.00, precision of 0.89–0.96, F_1_ score of 0.88–0.90, and area under the curve (AUC) of 0.85–0.98. However, the morphology of the BCG signal is highly dependent on the measurement device [7] and also on the location and setting of the sensors.

BCG sensors are expected to be used outside hospitals owing to their ease of use; thus, it is important to consider their practical use. Although a sheet-type sensor can be easily introduced, sensor dislocation may easily occur in such uncontrolled environments. Additionally, because people usually move around while sleeping, the BCG sensor is not always in the best position. In previous studies that detected AF using sheet-type sensors, BCG sensors were frequently placed under the participant’s chest, a location that was considered to show a large signal. However, to the best of our knowledge, there have been no reports on performance measured at different locations. In this study, we aimed to detect AF using a piezoelectric rubber sheet BCG sensor. We recorded the BCG simultaneously using multiple sensors and evaluated the differences in AF detection performance according to the location of the sensors. Furthermore, we found that combining data from multiple sensors contributed to improved performance.

## 2. Materials and Methods

### 2.1. Study Participants

A total of 84 adults were enrolled in this study, comprising 10 healthy volunteers and 74 patients who were referred to the Institute of Science Tokyo Hospital for the treatment of cardiovascular diseases. Healthy participants were defined as those with no medical history or medications. This study was approved by the Ethics Committee of the Institute of Science Tokyo (No. M2022-091). All measurements were performed after obtaining written informed consent and in accordance with the institutional regulations.

### 2.2. Recording of BCG

**Figure 1:**
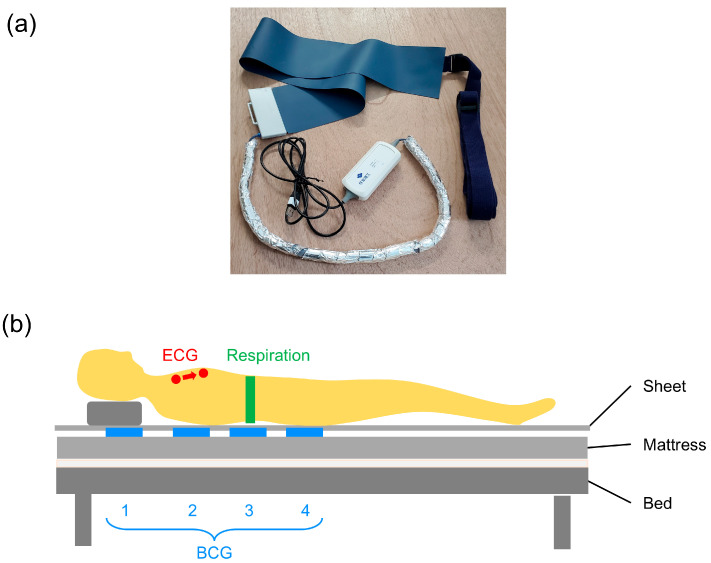
Placement of BCG and other sensors. (**a**) The BCG sensor used in this study. (**b**) Four BCG sensors (blue) were placed between the mattress and the disposable sheet. The bottom edges of the BCG sensors were aligned with 0, 25, 45, or 65 cm down from the base of the head. ECG was recorded using adhesive electrodes placed at the participant’s chest (red), and respiration signal was recorded using a belt-type sensor which measured the changes in chest diameter resulting from breathing (green). BCG, ballistocardiogram; ECG, electrocardiogram.

The BCG was recorded using a piezoelectric rubber sheet sensor (Sumitomo Riko, Tokyo, Japan) (Figure 1a). The sensor size was 947 × 112 × 0.6 mm (length × width × thickness), the sampling rate was 500 Hz, and 16-bit quantization was performed. Four sensors were placed between the sheet and the mattress on the bed. The bottom edges of the sensors were placed 0, 25, 45, and 65 cm from the base of the head (BCG1, BCG2, BCG3, and BCG4 sensors). The signals from the four sensors were recorded simultaneously for at least 5 min for all participants at rest in the supine position (Figure 1b).

### 2.3. Principle of the Piezoelectric Rubber Sheet Sensor

Piezoelectric rubber sheet sensors operate on the basis of the piezoelectric effect and generate an electric charge in response to mechanical stress [8,16]. Specifically, it detects vibrations generated by the cardiac activity, respiration, and body movement of an individual lying on it. The electric charge is converted into a digital signal and transmitted to a computer [17].

### 2.4. Recording of ECG and Respiration Signals

We recorded ECG and respiration signals along with BCG recordings because the BCG sensor detected vibrations from both cardiac and respiratory activities. ECG signals were recorded using PowerLab and FE132 Bioamp (AD Instruments, Nagoya, Japan), and respiration signals were recorded using PowerLab and a respiratory belt transducer (TN1132/ST Respiratory Belt, AD Instruments). The recorded signals were analyzed using LabChart Pro software (v7.3.8, AD Instruments). The sampling rates of the ECG and respiration signals were 1000 Hz, and were downsampled to 500 Hz before analysis.

### 2.5. Preprocessing Data

The recorded data were divided into 32.768 s blocks, with sliding blocks every 4.096 s. As the minimum recording time was 5 min, we used 66 blocks per participant for the analysis. The BCG amplitude ranged from 0 to 65,535 in anonymous units.

First, the mean and standard deviation of the BCG amplitude were calculated for each block, and the BCG amplitude was standardized by subtracting the mean and dividing by the standard deviation.

Subsequently, a process was performed to identify and remove external noise. We applied two methods to identify external noise. The first method applies a threshold to the raw signal. We measured the external noise level owing to body movement in preliminary measurements and found that the BCG amplitude exceeded 34,000 in the presence of body motion. Therefore, we explored and optimized the threshold value around 34,000. The second method involves standardizing the data and selecting noise based on variations.

**Figure 2:**
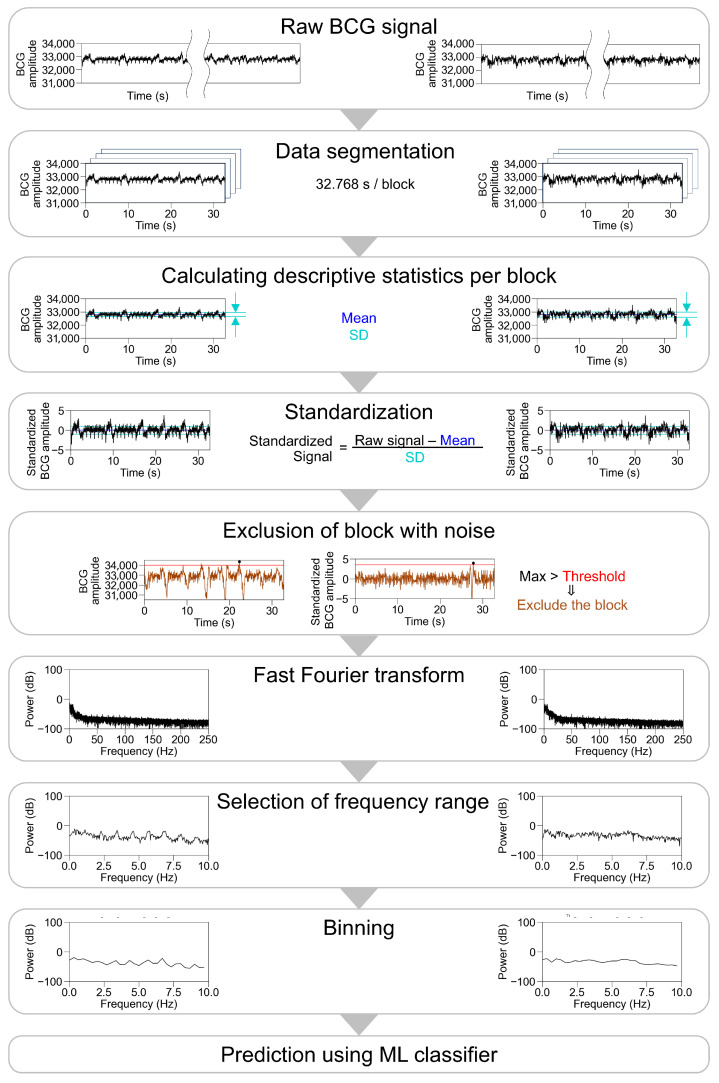
Pipeline and the representative traces during preprocessing of BCG waveform. Representative traces in participants with sinus rhythm are shown on the left panel, and those in participants with atrial fibrillation are shown on the right panel, excluding the part of the exclusion of block with noise. For the exclusion of block with noise, a threshold was applied to the raw signal (left) or the standardized signal (right). After a fast Fourier transform, multiple settings were examined for frequency ranges input into ML classifier, number of bins, type of ML classifier, and parameters of ML classifiers. BCG, ballistocardiogram; SD, standard deviation; ML, machine learning.

We then performed a fast Fourier transform (FFT) on the standardized BCG amplitude per block with a Hanning window using the Python library (SciPy 1.10.1) and used specific frequency ranges for the analysis. The examined cutoff frequencies are listed in Table 1 and the preprocessing pipeline is shown in Figure 2.

### 2.6. Data Composition

**Figure 3:**
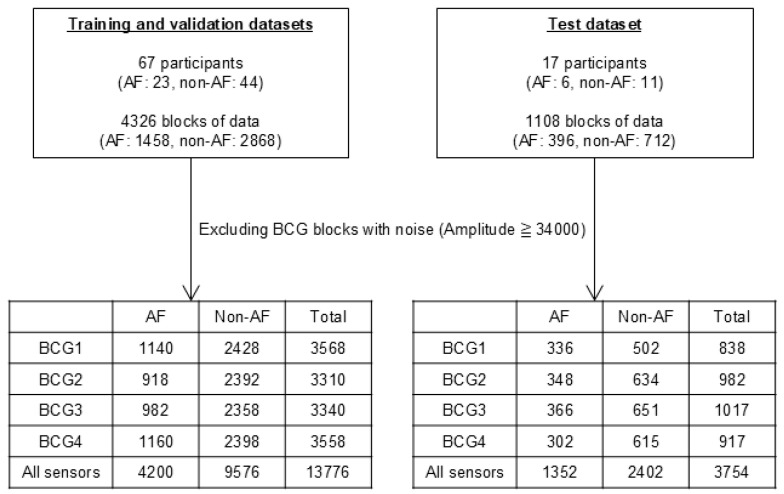
Number of datasets and participants and blocks in training, validation, and test datasets. The training and validation datasets were used for the training and optimization of the ML classifiers. The test dataset was used for the evaluation of classified results by the optimized ML classifiers. AF, atrial fibrillation; ML, machine learning.

A flowchart detailing the data composition is presented in Figure 3. A total of 5434 blocks from 84 participants were used in this study. We prepared three datasets: training and validation datasets comprising 4326 blocks from 67 participants and a test dataset comprising 1108 blocks from another 17 participants. After excluding blocks with body motion artifacts (BCG amplitude > 34,000), the training and validation datasets were used to train and optimize the ML classifiers. The test dataset was used to evaluate the results obtained using the optimized ML classifiers. The training, validation, and test datasets were derived from different participants to avoid overfitting. We used data from each of the four sensors separately and analyzed the combined BCG data from all the sensors. Thus, we used five datasets (BCG1, BCG2, BCG3, BCG4, and all sensor data).

### 2.7. Machine Learning Classifiers

Four types of ML classifiers, namely, the decision tree classifier (DT), random forest classifier (RF), logistic regression (LR), and AdaBoost classifier (ADA), were utilized to classify the transformed BCG signals into AF and non-AF groups. The ML classifiers were implemented using scikit-learn v1.3.0 with Python v3.11.4. We employed a stratified group five-fold cross validation (CV) and grid search to tune the hyperparameters. Specifically, we split the participants in the training and validation datasets into five groups while preserving the distribution of AF and non-AF in each group. Data from the same participant were never present in both the training and validation groups. We also examined whether changing frequency ranges input into ML classifiers and binning improved the ML classifiers. The power spectrum of the BCG signals below 250 Hz contained 8193 points of data between 0 and 250 Hz. We changed the cutoff frequencies and the number of bins, and optimized them for each classifier.

We selected the ML classifiers and other settings to maximize the mean value of accuracy during the grid search with five-fold CV for each sensor. The settings tested to optimize the ML classifiers are listed in Table 1. In addition, leave-one-participant-out CV using the best ML classifiers and settings was performed to assess generalization performance.

After optimization, the settings were fixed for each best ML classifier. The best ML classifiers and settings were used for evaluation using the test dataset and applied to datasets with different locations.

### 2.8. Statistical Analysis

Statistical analyses were conducted using the Python library statistical packages (pandas v1.5.3 and scikit-learn v1.3.0).

## 3. Results

### 3.1. Comparison of BCG Signals Between Participants with and Without AF

**Figure 4:**
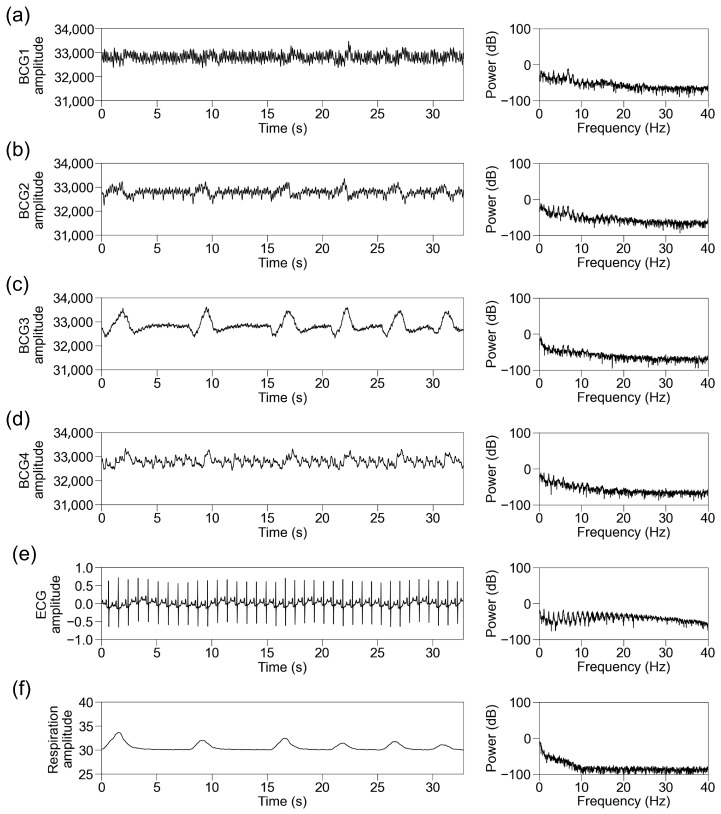
Representative traces in participants with sinus rhythm. Representative traces of BCG1 (**a**), BCG2 (**b**), BCG3 (**c**), BCG4 (**d**), ECG (**e**), and respiration signal (**f**) during one block (32.768 s). (Left panel) Raw wave for one block. (Right panel) Power spectrum below 40 Hz after fast Fourier transform. BCG, ballistocardiogram; ECG, electrocardiogram.

**Figure 5:**
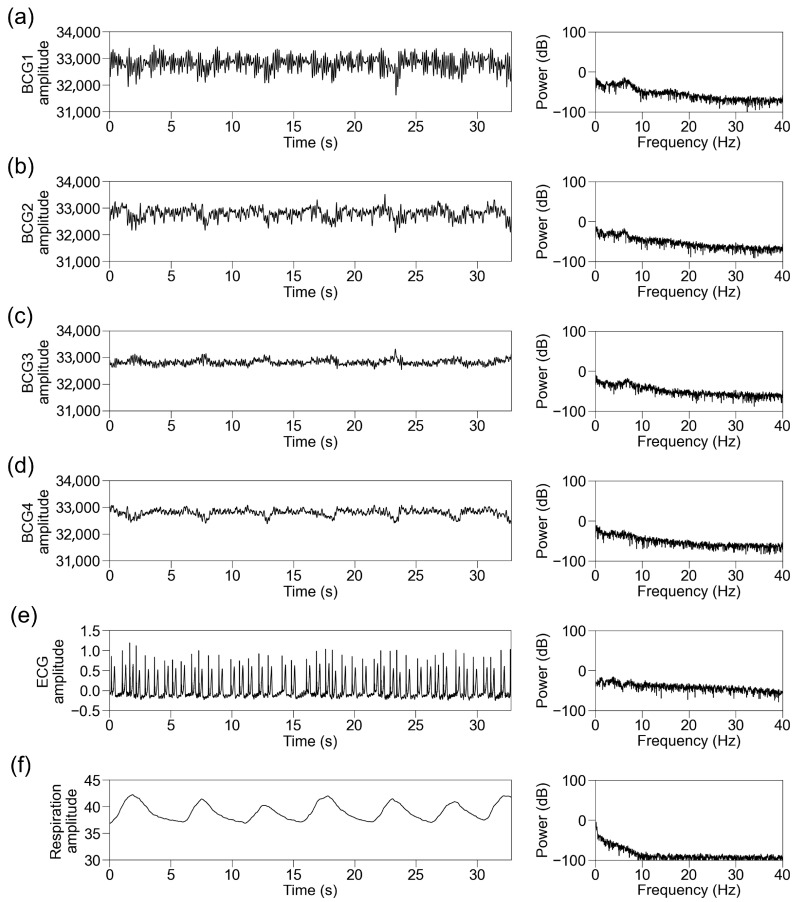
Representative traces in participants with atrial fibrillation. Representative traces of BCG1 (**a**), BCG2 (**b**), BCG3 (**c**), BCG4 (**d**), ECG (**e**), and respiration signal (**f**) during one block (32.768 s). (Left panel) Raw wave for one block. (Right panel) Power spectrum below 40 Hz after fast Fourier transform. BCG, ballistocardiogram; ECG, electrocardiogram.

We simultaneously recorded BCG signals from four sensors at different locations (BCG1, BCG2, BCG3, and BCG4), ECG signals, and respiration signals. Representative traces of the raw recorded signals and their power spectra in participants with sinus rhythm are shown in Figure 4. The raw BCG signals contained signals with periods matching those of ECG and respiration. We then standardized the data per block of 32.768 s and applied an FFT. The power spectrum of the ECG signal below 40 Hz showed the power of periodic frequency bands approximately every 1 Hz, which decreased as the frequency increased. The power spectrum of the respiratory signal showed power at a lower frequency. The power spectra of the BCG signals contained the power of the periodic frequency bands and lower frequency bands. The periodic power distribution was prominent below 10 Hz, slightly visible at 10–20 Hz, and almost invisible above 20 Hz; thus, we focused on the power spectrum below 10 Hz. Among the signals from the four sensors, it was observed most obviously in BCG2. The representative traces in participants with AF showed disappearance of the periodic power distribution of the ECG and BCG signals (Figure 5).

### 3.2. Optimization of ML Classifiers

We then attempted to classify the power spectra of the BCG signals into AF and non-AF groups using four types of ML classifiers (DT, RF, LR, and ADA). We used 4326 blocks of data from 67 participants for optimization. The background characteristics of the participants and BCG amplitudes of the blocks are shown in Table 2.

We optimized the ML classifiers to maximize the mean value of accuracy during the grid search five-fold CV. After excluding blocks containing motion artifacts, 3310–3568 blocks of data from sensors BCG1–BCG4 were used for this process. A total of 13,776 blocks of data from all sensors were also input into the ML classifiers, as well as single sensors (Figure 3).

Parameter optimization was performed for all the classifiers using a grid search. The mean values of accuracy during the five-fold CV with optimized settings per BCG sensor and ML classifier are listed in Table 3. The accuracy with the optimized settings depended more on the BCG sensor than on the type of ML classifier. The best classifiers for each sensor were ADA for BCG1 (accuracy: 0.89), ADA for BCG2 (0.91), RF for BCG3 (0.83), DT for BCG4 (0.76), and RF for all sensors (0.82).

The optimized parameters of the best ML classifiers for each BCG sensor are listed in Table 4. In the best ML classifiers, signals below 40 Hz were used, and the data were binned into a lower number of frequency bands. However, the contribution of low-frequency power cutting was not consistent across models.

We fixed the settings for each best ML classifier to those listed in Table 4 in the analysis after optimization. For example, when we used ADA for BCG2, we used BCG signals of 1–10 Hz and binned them into 30 frequency bands; the max_depth was 7 and the learning_rate was 0.9 in ADA for BCG2.

### 3.3. Removal of External Noise

**Figure 6:**
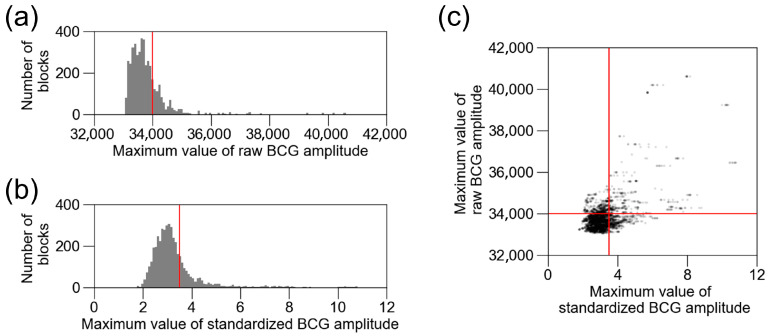
Distribution of raw BCG amplitude and standardized BCG amplitude. (**a**) Distribution of raw BCG amplitude in training and validation datasets of BCG2. Red line (34,000) is the threshold for excluding BCG blocks with noise. (**b**) Distribution of standardized BCG amplitude in training and validation datasets of BCG2. Red line is the threshold for excluding the same number of blocks as raw BCG amplitude > 34,000. (**c**) Standardized BCG amplitude was plotted against raw BCG amplitude. Red lines are the same as (**a**,**b**). BCG, ballistocardiogram.

To remove external noise, we tested two strategies: raw amplitude-based and standardized amplitude-based thresholds. Figure 6a shows the distribution of the raw amplitude, and Figure 6b shows the standardized amplitude in the training and validation datasets of BCG2. Both histograms show a right-skewed distribution. We excluded blocks with a BCG amplitude > 34,000 as the initial value, based on preliminary measurements. The threshold for a raw BCG amplitude > 34,000 excluded 23% of the data. When the standardized BCG amplitude-based threshold was set to exclude the same portion of data, the threshold was 3.4934. The distribution of the excluded data differed between the raw and standardized thresholds (Figure 6c). Table 5 shows the changes in the number of included data and accuracy in the training and validation datasets. The exclusion of raw BCG amplitudes > 34,000 resulted in the highest accuracy. Therefore, we decided to continue using this threshold.

### 3.4. Testing of Optimized ML Classifiers

We also performed a leave-one-participant-out CV using the best ML classifiers and settings to assess their generalization performance. The ADA for BCG2 exhibited the highest mean and lowest standard deviation among the models, followed by the ADA for BCG1, RF for BCG3, and DT for BCG4. The RF for all sensors showed a mean higher than the DT for BCG4, and a standard deviation as low as the ADA for BCG2 (Table 4).

**Figure 7:**
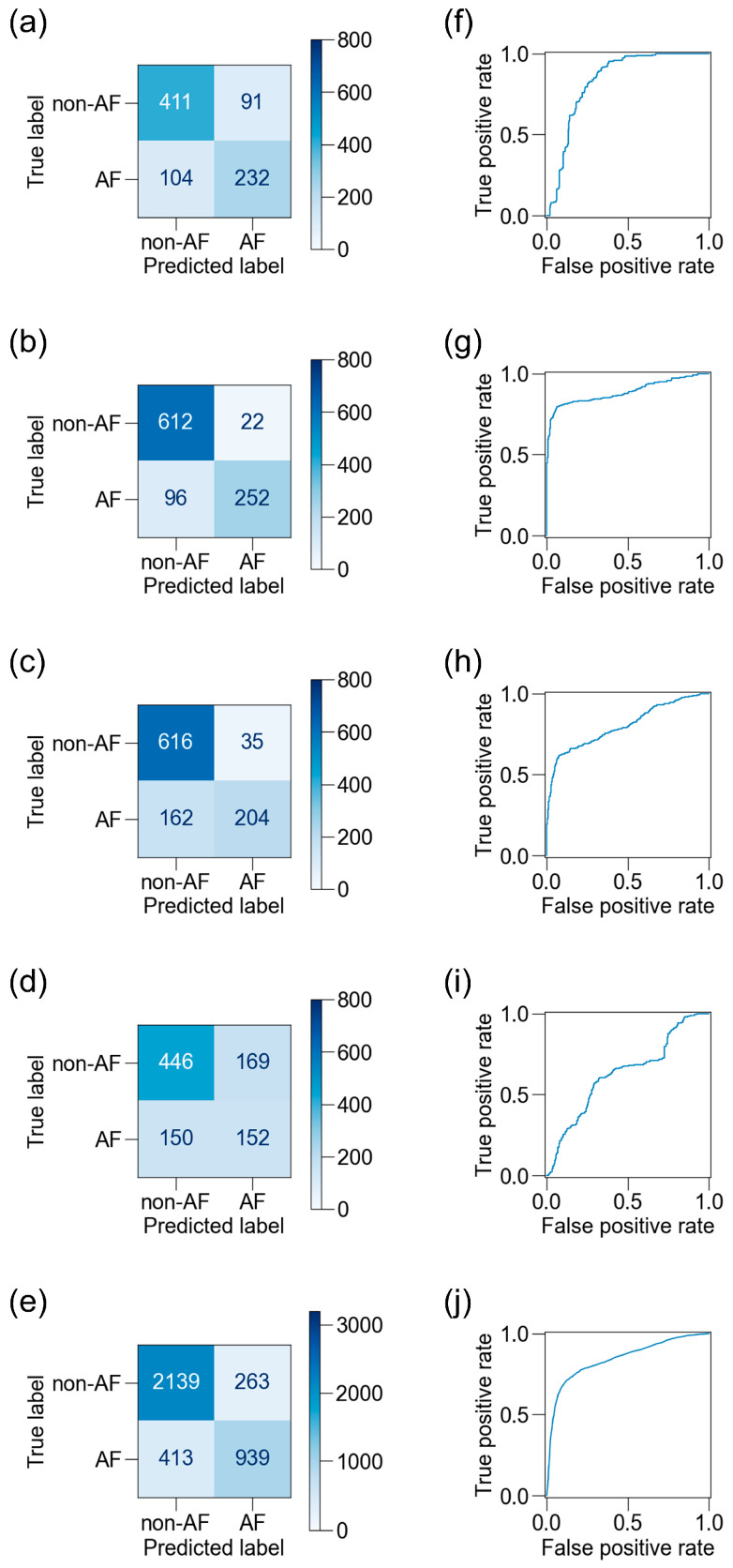
Confusion matrices and receiver operating characteristic (ROC) curves for BCG sensors. Confusion matrices on the test dataset are shown for the best ML classifiers for BCG1 (**a**), BCG2 (**b**), BCG3 (**c**), BCG4 (**d**), and a combination of all four sensors (**e**), and the corresponding ROC curves are shown (**f**–**j**). The accuracy was highest and the area under the ROC curve was largest in BCG2. BCG, ballistocardiogram; ML, machine learning.

We then used another 1108 blocks of data from the 17 participants to test the best ML classifiers. The participants’ background characteristics are presented in Table 2. After excluding noisy data, 838–1017 blocks of data from the four sensors and 3754 blocks of data from all sensors were used for evaluation (Figure 3). The confusion matrices and receiver operating characteristic (ROC) curves for the best classifiers are shown in Figure 7, and the scores are listed in Table 4. The ADA for BCG2 had the best scores: accuracy, 0.88; recall, 0.72; specificity, 0.97; precision, 0.92; F_1_ score, 0.81; and AUC, 0.89.

### 3.5. Influence of Location of BCG Sensors on Detection of AF

We applied the five best classifiers—derived from BCG1–4 sensors and combined all sensor data—to test the datasets acquired from different sensors to evaluate the robustness of the classifier to sensor location shifts (Table 6). The ADA for BCG2 generally obtained higher scores than the other classifiers, and its accuracy for the BCG1 dataset remained high (0.92). The accuracy declined when the model was applied to the test datasets recorded by the BCG3 and BCG4 sensors (0.73 and 0.78, respectively). These results suggest that the ADA for BCG2 was robust against the shift in the sensor toward the head direction, and to some extent robust against the shift toward the lumbar direction. No other ML classifier, including the RF for all sensors, showed higher robustness than the ADA for BCG2.

**Figure 8:**
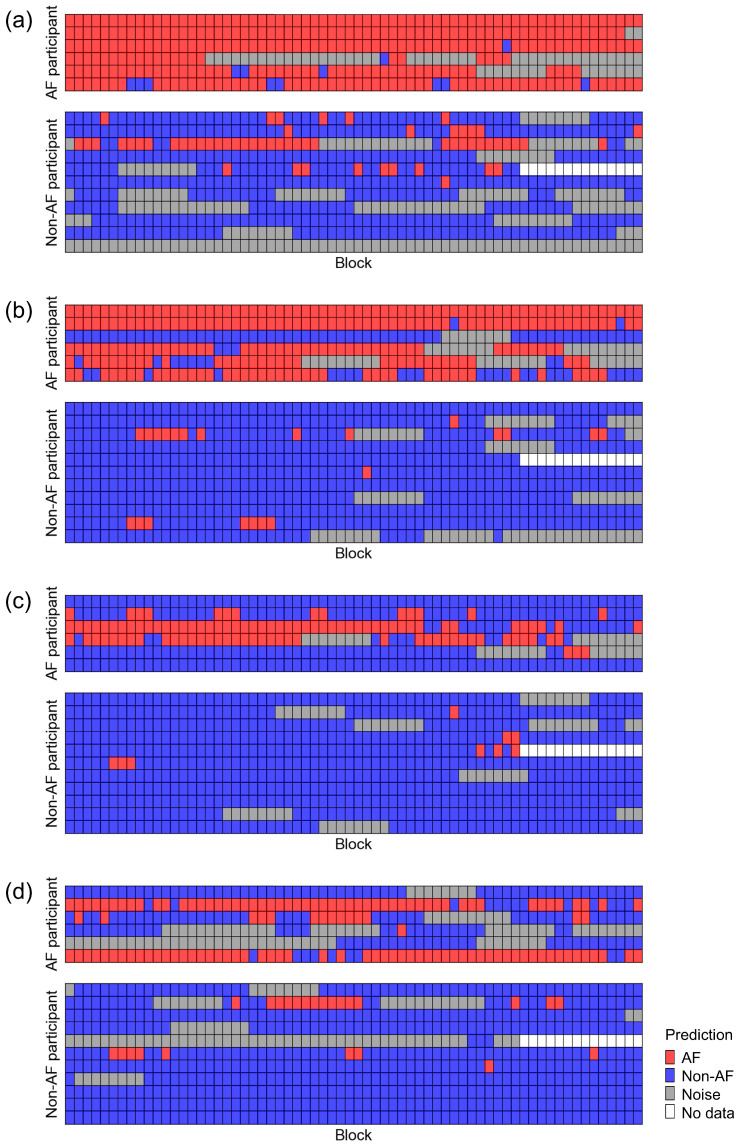
Classification results of the test dataset using the best classifier for BCG2. Datasets recorded using BCG1 (**a**), BCG2 (**b**), BCG3 (**c**), and BCG4 (**d**) sensors were classified using the best classifier for BCG2 (ADA for BCG2). A test dataset consisted of 6 AF participants (upper panel) and 11 non-AF participants (lower panel). AF, atrial fibrillation; BCG, ballistocardiogram.

**Figure 9:**
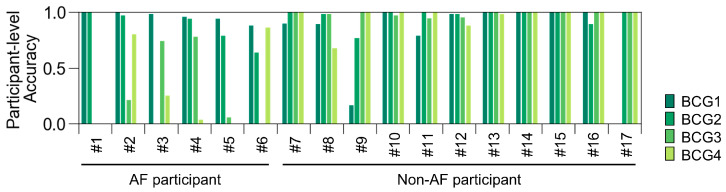
Participant-level accuracy of the test dataset using the best classifier for BCG2. Participant-level accuracy was calculated as (number of blocks predicted as AF)/((number of blocks predicted as AF) + (number of blocks predicted as non-AF)) in AF participants; and (number of blocks predicted as non-AF)/((number of blocks predicted as AF) + (number of blocks predicted as non-AF)) in non-AF participants. AF, atrial fibrillation; BCG, ballistocardiogram.

Because the BCG data were recorded for 5 min, they consisted of 66 blocks for each participant. We checked the predictions of ADA for BCG2 for all BCG data for each participant (Figure 8). The prediction accuracy for the BCG2 dataset was almost correct, except for one participant in the AF group who showed some false negative blocks. In the BCG1 dataset, the number of false positive blocks increased, whereas the number of false negative blocks decreased slightly. In the BCG3 and BCG4 datasets, the number of false negative blocks increased, resulting in a reduced recall. However, the number of false positive blocks did not increase in the BCG 3 and BCG4 datasets. Therefore, the ADA for BCG2 maintained high precision for all BCG data. Participant-level accuracies are summarized in Figure 9. BCG2 data exhibited low accuracy (0.00) only in participant #3, who was of relatively small stature (140 cm tall and weighing 37 kg). In addition, BCG3 and BCG4 data in participants #1 and #5 showed low accuracy. We found that the raw waveforms of BCG3 and BCG4 in these participants had very small signals in phases matched with ECG.

### 3.6. Combining BCG Data Recorded at Multiple Locations

**Figure 10:**
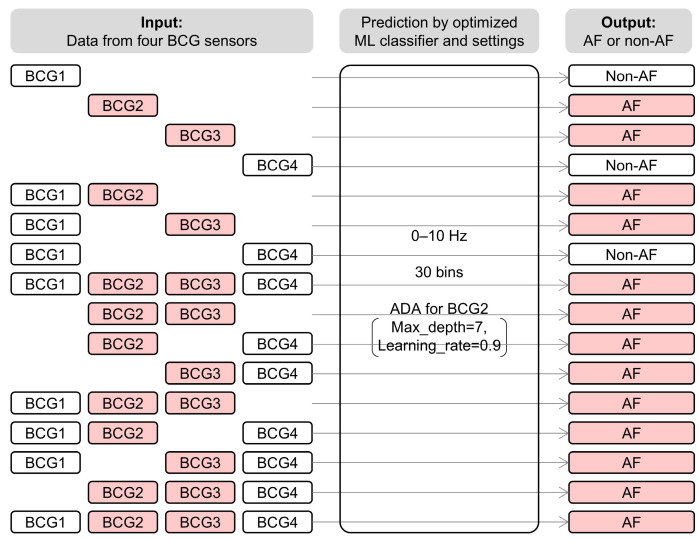
System architecture of combination analysis of BCG data recorded at multiple locations. All data were analyzed using ADA for BCG2 with the best settings. The block was classified as AF if any one prediction from the data was AF. For example, when the model classified data from BCG2 and BCG3 as AF, all combinations including BCG2 or BCG3 were predicted as AF. BCG, ballistocardiogram; ML, machine learning; AF, atrial fibrillation; ADA, AdaBoost classifier.

Finally, we examined whether combining data from multiple BCG sensors could improve prediction accuracy. We combined two to four predictions by ADA for BCG2 from the same block of the test dataset and classified the block as AF if any one of the predictions was AF (Figure 10). The combinations and scores are presented in Table 7. The highest accuracy was obtained with the combination of the BCG1 and BCG2 data, which exhibited an accuracy of 0.92, recall of 0.98, specificity of 0.89, precision of 0.84, and F_1_ score of 0.90. The combined model reduced the number of blocks excluded owing to noise, resulting in the inclusion of 1013 blocks in total (AF: 367 blocks, non-AF: 646 blocks). It showed a higher recall than the assessment based solely on BCG1 or BCG2 data. Its accuracy and F_1_ score were as high as those of the assessment based on BCG1 data and higher than those based on BCG2 data.

## 4. Discussion

In this study, we recorded the BCG using a piezoelectric rubber sheet sensor placed on a bed and developed an AF detection algorithm using ML classifiers. The location of the sensor affected the waveforms of the BCG and AF detection results. The optimized AdaBoost classifier for the sensor placed 25 cm from the head position showed the best classification results, with an accuracy of 0.88, recall of 0.72, specificity of 0.97, precision of 0.92, and F_1_ score of 0.81. The classifier was robust against the shift in the sensor location toward the head direction, and to some extent robust against the shift toward the lumbar direction. Combining data from the sensors at 0 and 25 cm from the head position reduced data exclusion due to noise and achieved an accuracy of 0.92, recall of 0.98, specificity of 0.89, precision of 0.84, and F_1_ score of 0.90.

We performed an FFT of the BCG signals and focused on the power spectrum below 10 Hz. The power spectrum indicated a pattern containing waves of approximately 1 Hz and their multiples in non-AF participants but not in AF participants. We speculated that the pattern reflected the regularity of the sinus rhythm because the regular signals at approximately 1 Hz matched the pulse wave of the normal sinus rhythm. We then adopted ML classifiers, focusing on the differences in the power spectrum patterns. A similar analytical approach has been employed in previous investigations that used different devices to detect AF. One study using a blood pressure monitor quantified the noise around the heart rate frequency [18]. Another study using the smartphone-derived seismocardiography and gyrocardiography signals calculated the harmonic-to-noise ratio [19]. Regarding studies using BCG to detect AF, one study used a feature called “periodicity” [15]. While all these studies, including ours, exploited the regularity of the pulse wave during sinus rhythm, the specific approaches to feature extraction varied.

In the last decade, several studies have been conducted to detect AF using the BCG. Related works are presented in Table 8. Our ADA for the BCG2 classifier showed lower recall, slightly lower accuracy, and lower F_1_ scores than those of other studies using BCG. We speculate that this is because of the many false negatives and the low proportion of AF in the test dataset, which means that one false negative had a significant effect. In contrast, ADA for BCG2 had a higher specificity and comparable precision because there were few false positives. Combination analysis using BCG1 and BCG2 data improved recall, F_1_ score, and accuracy, but decreased precision. Other modalities, including photoplethysmography [20], mechanocardiography [19], acoustic sensing [21], and millimeter wave radar [22], have also been developed for AF detection (Table 8). The performance in our study appears lower than that in some of these studies. Although sheet-type sensors can be used in a less invasive way, they are more susceptible to external noise than contact-type sensors. In addition, the differences in the volume of training data might affect the results, and the differences in testing models might also influence the performance. Direct comparison with simultaneous recording in the same participants regarding the performance and user experience may help the user to select an appropriate method in the future. The prevalence of AF increases with age, and the overall prevalence is projected to reach 1.09% by 2050 in Japan [23]. If we screen for AF without selecting a high-risk population in the real world, we may encounter many false positives. The ADA for BCG2 is unlikely to produce false positives; thus, it is considered suitable for use in such situations. Conversely, it is appropriate to use a combination analysis using BCG1 and BCG2 data or algorithms in other studies in populations with a high incidence of AF to avoid the underdetection of AF.

We optimized the classifier by changing the low and high cutoff frequencies, number of bins, and the parameters of the ML classifiers, in addition to the selection of the type of ML classifier and the sensor location. Among them, the sensor location and high cutoff frequency had a significant effect. The BCG2-derived training dataset and lower high cutoff frequency contributed to higher accuracy. The BCG2 sensor was located under the participant’s chest at heart level, and its data contained abundant periodic frequency bands approximately every 1 Hz in non-AF participants. In contrast, BCG3 and BCG4 sensors were located at the lumbar lordosis level and the sacral kyphosis level, respectively, and their data contained few periodic frequency bands approximately every 1 Hz. They were located near the organs that moved during respiration, such as the diaphragm, and contained frequency bands synchronized with respiration. We speculate that the higher accuracy in the BCG2-derived training dataset was due to the abundant periodic frequency bands approximately every 1 Hz in non-AF participants in the BCG2 data. We considered that it might be preferable to remove frequency bands synchronized with respiration and examined multiple low cutoff frequencies; however, the contribution of low-frequency power cutting was not consistent. We thus consider that the influence of respiration is negligible. The periodic frequency bands were prominent below 10 Hz and decreased in the higher-frequency range. Higher frequency bands are inefficient for classification; therefore, cutting them would make it easy for the classifier to detect the differences between AF and non-AF.

We evaluated the influence of the BCG sensor location by applying the best ML classifier to the test datasets recorded at different locations. This is the first study to compare the scores for AF detection among BCG data simultaneously recorded at different locations. The positional relationship between the BCG sensor and the participant varied by study and product. In other studies that recorded the BCG in patients with AF in the supine position, piezoelectric sheet-type sensors were placed between the mattress and the participant’s chest [10,14] or under the mattress [13], a pressure-sensitive mat-type sensor was integrated into a mattress [15], or a microelectromechanical accelerometer-based sensor was attached under the bed [12]. In this study, four BCG sensors were positioned within a 65 cm range from the head to the lumbar region. The optimized ML model (ADA for BCG2) demonstrated a certain consistent accuracy across all the four sensor locations. Notably, the AF detection accuracy remained unchanged when the sensor was shifted toward the head direction, whereas the number of false negatives increased when the sensor was moved toward the lumbar direction. False positives were rarely observed, and precision was maintained even when the sensor placement deviated from the optimal chest position. These findings suggest that the proposed ML algorithm remains robust and clinically applicable even when the BCG sensors are not optimally positioned.

Combination analysis using sensors at 0 and 25 cm from the head position reduced exclusion due to noise and improved recall. This is because the classification result of the ADA for BCG2 had relatively more false negatives and fewer false positives, and our combination algorithm classified the block as AF if any one of the predictions was AF. This usage contributed to enhancing the robustness against noise and minimizing the underdetection of AF. Expanding the options for combining multichannel data could potentially improve accuracy. This study demonstrated that signals differed depending on the sensor location, even when they were recorded simultaneously. Although our analysis simply combined the predictions based on multichannel data, a more complex combination might utilize the data more effectively. We speculate that if the algorithm could predict the sensor location or evaluate the signal validity, it could select signals more appropriately or combine them with suitable weightings. The application of more advanced techniques such as deep learning to this issue is anticipated in the future.

This study has several limitations. First, the sample size was small. Second, data were collected from a single facility. As with other studies, external testing should be conducted in the future to assess generalization ability. Third, the ML classifiers were evaluated using data recorded under controlled conditions. Body movements, body orientation, and postures other than the supine position may affect the performance of ML classifiers. We need to evaluate this in participants lying freely in bed. As a next step, external validation in a realistic environment is required to use the proposed algorithm in practice.

## 5. Conclusions

We established ML algorithms to detect AF from BCG using piezoelectric rubber sheet sensors at four locations. The optimized model for BCG 25 cm from the head showed the highest accuracy (0.88). The model was robust against shifts in the sensor toward the head and lumbar directions. A combined assessment using multiple sensors improved performance.

## Figures and Tables

**Table 1:** Settings used to optimize the ML classifiers.

Variables			Configurations
Frequency ranges input into ML classifier (Hz)	High cutoff frequency		10, 20, 40, 250
	Low cutoff frequency		0, 0.5, 0.7, 1.0, 1.2
Number of bins	3, 5, 10, 30, 40, 50, 100, 150		
Parameters	DT	max_depth	1, 3, 5, 7, 10, 15
	RF	max_depth	1, 3, 5, 7, 10, 15, 20, 25, 30, 40
	LR	C	10^−8^, 10^−6^, 10^−4^, 10^−2^, 1, 10^2^, 10^4^, 10^6^, 10^8^
	ADA	learning_rate	1.0, 0.9, 0.8
		max_depth	1, 2, 3, 4, 5, 6, 7, 8
		Estimator	DT

DT, decision tree classifier; RF, random forest classifier; LR, logistic regression; ADA, AdaBoost classifier.

**Table 2:** Background characteristics and BCG amplitude in training, validation, and test datasets.

	Training and Validation Datasets		Test Dataset	
	AF	Non-AF	AF	Non-AF
Number of participants, *n*	23	44	6	11
Number of blocks, *n*	1458	2868	396	712
Age (years)	75 ± 11	66 ± 20	75 ± 11	61 ± 28
Male, *n* (%)	15 (65)	27 (61)	5 (83)	6 (55)
Height (cm)	163 ± 10	164 ± 9	165 ± 13	159 ± 8
Weight (kg)	61 ± 15	59 ± 11	64 ± 15	54 ± 8
Body mass index	23 ± 4	22 ± 3	23 ± 3	21 ± 2
Systolic blood pressure (mmHg)	118 ± 20	120 ± 16	112 ± 8	124 ± 20
Diastolic blood pressure (mmHg)	76 ± 11	75 ± 11	74 ± 11	75 ± 9
Heart rate (bpm)	70 ± 10	64 ± 14	82 ± 10	67 ± 16
BCG1				
Maximum value of one block	33,833 ± 736	33,690 ± 380	33,712 ± 603	33,962 ± 800
Mean value of one block	32,806 ± 6	32,806 ± 4	32,806 ± 3	32,806 ± 6
Minimum value of one block	31,680 ± 839	31,824 ± 585	31,818 ± 1001	31,658 ± 796
BCG2				
Maximum value of one block	34,032 ± 1008	33,687 ± 568	33,694 ± 502	33,672 ± 400
Mean value of one block	32,794 ± 5	32,794 ± 5	32,794 ± 4	32,794 ± 4
Minimum value of one block	31,302 ± 1250	31,745 ± 712	31,531 ± 1156	31,784 ± 337
BCG3				
Maximum value of one block	34,117 ± 2006	33,703 ± 849	33,679 ± 1178	33,570 ± 357
Mean value of one block	32,829 ± 11	32,830 ± 6	32,830 ± 4	32,830 ± 5
Minimum value of one block	31,509 ± 2174	31,950 ± 763	31,688 ± 2039	31,961 ± 525
BCG4				
Maximum value of one block	33,790 ± 712	33,707 ± 533	33,869 ± 539	33,525 ± 339
Mean value of one block	32,803 ± 8	32,802 ± 6	32,802 ± 6	32,802 ± 4
Minimum value of one block	31,851 ± 1020	32,067 ± 525	31,799 ± 818	32,239 ± 257

Data are presented as mean ± standard deviation. BCG, ballistocardiogram.

**Table 3:** Mean accuracy on validation dataset per BCG sensor and ML classifier.

	DT	RF	LR	ADA
BCG1	0.83	0.85	0.88	0.89
BCG2	0.85	0.90	0.87	0.91
BCG3	0.81	0.83	0.82	0.82
BCG4	0.76	0.73	0.74	0.74
All sensors	0.78	0.82	0.81	0.81

The highest mean values of accuracy during the grid search five-fold cross validation per BCG sensor and ML classifier are presented. BCG, ballistocardiogram; ML, machine learning; DT, decision tree classifier; RF, random forest classifier; LR, logistic regression; ADA, AdaBoost classifier.

**Table 4:** Best ML classifiers and settings for each BCG sensor.

	BCG1	BCG2	BCG3	BCG4	All Sensors
Best ML classifier	ADA	ADA	RF	DT	RF
Best settings					
Frequency ranges input into ML classifier (Hz)	1.2–20	0–10	1–10	0.5–40	1–10
Number of bins	10	30	50	3	100
Parameters	max_depth = 1, learning_rate = 0.8	max_depth = 7, learning_rate = 0.9	max_depth = 10	max_depth = 3	max_depth = 30
Accuracy during the five-fold CV on validation dataset ^1^	0.89	0.91	0.83	0.76	0.82
Accuracy during the leave-one-participant-out CV on validation dataset ^2^	0.83 ± 0.29	0.86 ± 0.24	0.79 ± 0.30	0.75 ± 0.34	0.78 ± 0.24
Scores on test dataset					
Accuracy	0.61	0.88	0.81	0.75	0.82
Recall	0.32	0.72	0.56	0.42	0.69
Specificity	0.81	0.97	0.95	0.92	0.89
Precision	0.53	0.92	0.85	0.71	0.78
F_1_ score	0.40	0.81	0.67	0.53	0.74
AUC	0.66	0.89	0.80	0.79	0.84

^1^ is expressed as mean. ^2^ is expressed as mean ± standard deviation. ML, machine learning; BCG, ballistocardiogram; ADA, AdaBoost classifier; RF, random forest classifier; DT, decision tree classifier; ML, machine learning; CV, cross validation; AUC, area under the curve.

**Table 5:** Change in number of included data and accuracy against threshold.

		Included Blocks, *n* (%)	Mean Accuracy During the Five-Fold CV on Validation Dataset
Raw BCG amplitude-based threshold			
	33,500	1502 (35)	0.87
	34,000	3310 (77)	0.91
	34,500	3971 (92)	0.84
	35,000	4177 (97)	0.84
	35,500	4217 (97)	0.84
	36,000	4249 (98)	0.85
Standardized BCG amplitude-based threshold			
	3	1988 (46)	0.87
	3.4934	3310 (77)	0.86
	4	3825 (88)	0.86
	5	4124 (95)	0.82
	6	4214 (97)	0.85

BCG, ballistocardiogram; CV, cross validation.

**Table 6:** Performance of ML classifiers applied to BCG data recorded at multiple locations.

Scores	ML Classifiers	Test Dataset			
		BCG1	BCG2	BCG3	BCG4
Accuracy	ADA for BCG1	**0.61**	0.62	0.68	0.69
	ADA for BCG2	0.92	**0.88**	0.73	0.78
	RF for BCG3	0.75	0.75	**0.81**	0.70
	DT for BCG4	0.63	0.70	0.65	**0.75**
	RF for all sensors	0.84	0.90	0.78	0.76
Recall	ADA for BCG1	**0.32**	0.04	0.11	0.25
	ADA for BCG2	0.96	**0.72**	0.29	0.41
	RF for BCG3	0.78	0.84	**0.56**	0.65
	DT for BCG4	0.73	0.97	0.49	**0.42**
	RF for all sensors	0.90	0.83	0.47	0.58
Specificity	ADA for BCG1	**0.81**	0.94	1.00	0.90
	ADA for BCG2	0.88	**0.97**	0.99	0.96
	RF for BCG3	0.74	0.70	**0.95**	0.73
	DT for BCG4	0.57	0.54	0.75	**0.92**
	RF for all sensors	0.79	0.94	0.96	0.85
Precision	ADA for BCG1	**0.53**	0.29	0.93	0.56
	ADA for BCG2	0.85	**0.92**	0.92	0.84
	RF for BCG3	0.66	0.60	**0.85**	0.54
	DT for BCG4	0.53	0.54	0.52	**0.71**
	RF for all sensors	0.75	0.88	0.88	0.65
F_1_ score	ADA for BCG1	**0.40**	0.07	0.20	0.35
	ADA for BCG2	0.90	**0.81**	0.44	0.55
	RF for BCG3	0.72	0.70	**0.67**	0.59
	DT for BCG4	0.61	0.69	0.50	**0.53**
	RF for all sensors	0.82	0.86	0.61	0.61
AUC	ADA for BCG1	**0.66**	0.60	0.87	0.61
	ADA for BCG2	0.95	**0.89**	0.75	0.82
	RF for BCG3	0.82	0.84	**0.80**	0.73
	DT for BCG4	0.69	0.78	0.68	**0.79**
	RF for all sensors	0.90	0.94	0.86	0.71

The scores for the test dataset are shown. Data recorded at the original location are indicated in bold. ML, machine learning; BCG, ballistocardiogram; ADA, AdaBoost classifier; RF, random forest classifier; DT, decision tree classifier; AUC, area under the curve; CV, cross validation.

**Table 7:** Combination analysis of BCG data recorded at multiple locations.

BCG Data Used for Analysis	Included Blocks, *n* (%)	Scores				
		Accuracy	Recall	Specificity	Precision	F_1_ Score
1	838 (76)	0.92	0.96	0.88	0.85	0.90
2	982 (89)	0.88	0.72	0.97	0.92	0.81
3	1017 (92)	0.73	0.29	0.99	0.92	0.44
4	917 (83)	0.78	0.41	0.96	0.84	0.55
1, 2	1013 (91)	0.92	0.98	0.89	0.84	0.90
1, 3	1025 (93)	0.92	0.95	0.90	0.84	0.89
1, 4	1057 (95)	0.90	0.92	0.88	0.80	0.86
2, 3	1069 (96)	0.91	0.83	0.96	0.91	0.87
2, 4	1077 (97)	0.88	0.78	0.94	0.86	0.82
3, 4	1083 (98)	0.82	0.55	0.96	0.87	0.67
1, 2, 3	1069 (96)	0.92	0.99	0.89	0.83	0.90
1, 2, 4	1090 (98)	0.90	0.95	0.87	0.80	0.87
1, 3, 4	1083 (98)	0.91	0.96	0.88	0.81	0.88
2, 3, 4	1090 (98)	0.91	0.87	0.93	0.87	0.87
1, 2, 3, 4	1090 (98)	0.90	0.98	0.86	0.79	0.88

BCG, ballistocardiogram.

**Table 8:** Comparison to related works.

Study	Koivisto et al. (2019) [12]	Jiang et al. (2021) [13]	Cheng et al. (2022) [14]	Sandelin et al. (2025) [15]	Our Study	
					ADA for BCG2	Combination Analysis of BCG1 and BCG2
Signal	BCG	BCG	BCG	BCG	BCG	
Sensor	MEMS accelometer based sensor	Piezoelectric film sensor	Piezoelectric film sensor	Pressure-sensitive mat	Piezoelectric rubber sheet sensor	
Posture	Lying position	Lying position	Lying position	Supine position	Supine position	
Cohort size	20 AF and 15 healthy participants.	2000 (AF:1000, non-AF:1000) segments from 59 patients with PAF. 80% of segments were used for training, and 20% of segments were used for test.	4000 (AF:2000, non-AF:2000) segments from 59 patients with PAF. 80% of segments were used for training, and 20% of segments were used for test.	72 (AF: 32, SR: 40) participants for training, and 44 (AF: 22, SR: 22) participants for test.	3310 (AF: 918, non-AF: 2392) blocks from 67 participants for training, and 982 (AF: 348, non-AF: 634) blocks from 17 participants for test.	3310 (AF: 918, non-AF: 2392) blocks from 67 participants for training, and 1013 (AF: 367, non-AF: 646) blocks from 17 participants for test.
Best performance						
Accuracy	-	0.95	0.96	0.91	0.88	0.92
Recall	1.00	0.95	0.96	0.94	0.72	0.98
Specificity	0.93	0.94	0.96	0.86	0.97	0.89
Precision	-	0.94	0.96	0.93	0.92	0.84
F_1_ score ^1^	-	0.94	0.96	0.93	0.81	0.90
AUC	1.00	-	-	0.97	0.89	-
**Study**	**Pachori et al.** **(2024) [20]**	**Jafari Tadi et al.** **(2019) [19]**	**Liu et al.** **(2024) [21]**	**Zhao et al.** **(2024) [22]**		
Signal	PPG	MCG (SCG + GCG)	Acoustic sensing	Millimeter wave		
Sensor	Bedside monitor	Built-in accelerometer and gyroscope in smartphone	Built-in microphones and speakers in smartphone	Millimeter wave radar		
Posture	(Publicly available dataset)	Supine position	-	Lying position		
Cohort size	35 (AF:19, non-AF: 16) participants for 10-fold CV. 4200 samples were used for training, and 4200 samples were used for validation.	300 (AF: 150, SR: 150) participants for training, and 135 (AF:40, SR: 95) participants for test.	764 valid data pieces (AF: 27.9%, non-AF: 72.1%) from 20 participants for leave-one-participant-out CV.	5 s window with 1 s sliding × a total duration of 26.6 h data (arrhythmia: 20.7%, normal sinus rhythm: 79.8%) from 20 participants. 70% were used for training, 10% were used for validation, and 20% were used for test.		
Best performance						
Accuracy	0.99	0.95	0.93	0.97 ^2^		
Recall	0.99	0.90	0.87	0.92 ^2^		
Specificity	0.99	0.97	-	0.99 ^2^		
Precision	-	0.92	0.87	0.95 ^2^		
F_1_ score ^1^	0.99	0.96	0.87	0.93 ^2^		
AUC	1.00	-	-	1.00 ^2^		

^1^ If recall and precision were provided, we calculated the F_1_ score as 2 × (recall × precision)/(recall + precision). ^2^ Arrhythmia vs. normal sinus rhythm. MEMS, microelectromechanical system; BCG, ballistocardiography; PPG, photoplethysmography; MCG, mechanocardiography; SCG, seismocardiography; GCG, gyrocardiography; PAF, paroxysmal atrial fibrillation; SR, sinus rhythm; CV, cross validation; AUC, area under the curve.

## Data Availability

The original contributions of this study are included in this article. Further inquiries can be directed to the corresponding author.

## Abbreviations

The following abbreviations are used in this manuscript:

AF	Atrial fibrillation
BCG	Ballistocardiogram
ML	Machine learning
AUC	Area under the curve
FFT	Fast Fourier transform
DT	Decision tree classifier
RF	Random forest classifier
LR	Logistic regression
ADA	AdaBoost classifier
CV	Cross validation
ROC	Receiver operating characteristic
